# A New Algorithm for Visual Navigation in Unmanned Aerial Vehicle Water Surface Inspection

**DOI:** 10.3390/s25082600

**Published:** 2025-04-20

**Authors:** Jianfeng Han, Xiongwei Gao, Lili Song, Jiandong Fang, Yongzhao Tao, Haixin Deng, Jie Yao

**Affiliations:** 1 School of Information Engineering, Inner Mongolia University of Technology, Hohhot 010080, China; hanjianfeng@imut.edu.cn (J.H.); gxw374940468@163.com (X.G.); taoyongzhao163@163.com (Y.T.); dhaixin2022@163.com (H.D.); 15684137275@163.com (J.Y.); 2 Inner Mongolia Key Laboratory of Intelligent Perception and System Engineering, Hohhot 010080, China

**Keywords:** river tracking, visual navigation, UAV, semantic segmentation, attention mechanism

## Abstract

Water surface inspection is a crucial instrument for safeguarding the aquatic environment. UAVs enhance the efficiency of water area inspections due to their high mobility and extensive coverage. This paper introduces two UAV inspection methodologies for the characteristics of rivers and lakes, along with an efficient semantic segmentation algorithm, WaterSegLite (Water Segmentation Lightweight algorithm), for UAV visual navigation. The algorithm employs the UAV’s aerial perspective alongside a streamlined neural network architecture to facilitate rapid real-time segmentation of water bodies and to furnish positional data to the UAV for visual navigation. The experimental findings indicate that WaterSegLite achieves a segmentation accuracy (mIoU) of 93.81% and an F1 score of 95.44%, surpassing the baseline model by 2.7% and 2.23%, respectively. Simultaneously, the processing frame rate of this algorithm on the airborne device attains 28.27 frames per second, fully satisfying the requirements for real-time water surface inspection by UAVs. This paper offers technical assistance for UAV inspection techniques in aquatic environments and presents innovative concepts for the intelligent advancement of water surface inspection.

## 1. Introduction

Water surface environmental management, as one of the most important aspects of ecosystem protection, is critical to the overall environmental protection strategy as well as the maintenance of water bodies’ ecological balance. Water surface inspections are an important part of governance because they help to prevent the spread of water pollution and protect endangered aquatic flora and fauna by detecting and treating floating garbage, pollutants, and ecological anomalies in a timely manner. Traditional water surface inspection work primarily employs unmanned boat technology, which uses digital image processing technology to segment the Riverbank Line to enable the boat’s visual navigation [1,2,3], and then detects and identifies water surface garbage [4,5,6], and cleans it up using a cleaning device [7], robotic arm [8], and so on. However, the inspection method using an unmanned boat as a carrier is limited by the river environment and may be hampered by water terrain, obstacles, and other factors.

The UAV’s elevated vantage point allows for extensive water surface inspection coverage, enabling it to traverse a broader area in a brief timeframe, thereby markedly enhancing inspection efficiency. Simultaneously, its exceptional maneuverability allows UAVs to function in intricate aquatic settings, document the latitude and longitude of aerial images, identify garbage locations, and enhance cleanup efficiency. Abro et al. [9] presented a comprehensive examination of advancements in sensor fusion technology for 3D object detection, highlighting the critical contribution of multimodal sensors to enhancing detection precision and reliability. This fusion concept offers insights into multi-source information processing in intricate environments for unmanned aerial vehicles. Bayram et al. [10] proposed a machine learning approach utilizing UAV imagery to segment the shoreline, employing random forest and support vector machine techniques, respectively. Huang et al. [11] proposed a horizontal UAV inspection method for water surfaces to address scenarios where the UAV may inadvertently descend into the water during inspection. Rathinam et al. [12] utilized image processing techniques to segment Riverbank Line boundaries and employed GPS methods to monitor rivers. These methodologies offer technical assistance for river inspections via UAVs; however, there remains a deficiency of targeted strategies for lake inspections. Moreover, these methodologies emphasize large industrial-scale UAV solutions and high-performance computing systems, while optimized designs for small UAVs and edge devices remain insufficiently investigated. Consequently, we advocate for the deployment of small UAVs for the inspection of rivers and lakes, alongside visual navigation utilizing deep learning techniques.

Sequentially, algorithms that are appropriate for the detection of riverbank lines have been proposed as a result of the advancements in deep learning. The YOLOv7-BW method, which was proposed by Jin et al. [13], is a high-precision and efficient method for detecting dense small targets in remote sensing images. This method is based on an enhanced neural network model. The amount of model computation is significantly reduced by the use of U-Net for water body extraction, which employs a bottleneck structure in place of the conventional 3 × 3 convolution. This was proposed by An et al. [14]. The DeepLabV3 model was suggested for river extraction by Li et al. [15] and Su [16] et al. PSPNet is employed by Yin et al. [17] to detect shorelines in inland waters. The segmented graph is then processed using the Canny edge detection method. The Transformer [18] based self-attentive methods do not combine the advantages of convolution, which makes the model require more computational parameters. Because the model needs to capture global features, it typically has a high dependence on the amount of data and requires a large amount of training data and computational power to train and optimize the model, which means that they cannot be used in environments with limited resources or volume. Despite the high detection accuracy of these semantic segmentation-based algorithms in water shoreline detection, they are constrained by the computational resources of UAVs' onboard equipment. Consequently, it is challenging to meet the real-time inland waterways segmentation requirements of the onboard equipment. Therefore, more lightweight and efficient models are required for water segmentation. SA-BiSeNet, a two-branched downsampling structure that directly employs the BiSeNetV2 design, was proposed by Zhang et al. [19] for water surface segmentation. This structure includes spatial detail branches and semantic branches simultaneously. Yu et al. [20] developed feature attention modules that were integrated into each branch of the HRNet extractor. These modules were employed to improve the segmentation of small background content and fuzzy boundaries. The LiteSeg algorithm for semantic segmentation was proposed by Mara T. et al. [21]. This algorithm was developed using DeepLabV3 and utilized a deep separable convolution to replace traditional convolution. The algorithm also utilized long and short residual connections, which significantly enhanced the operational efficiency of the model. Although the lightweight semantic segmentation algorithm has a rapid processing speed that meets the real-time requirements of UAV waterways segmentation, it still encounters issues such as image processing lag, incorrect segmentation, and segmentation leakage when dealing with complex and variable water surface images. These issues directly impact the accuracy of water surface segmentation.

We require the UAV to execute visual navigation in order to facilitate autonomous river inspection. The UAV’s flight attitude is adjusted in accordance with the position information obtained from the water surface segmentation task. The water surface segmentation task is distinct from other target segmentation tasks in that the water body target is larger. The difficulty is in precisely segmenting the edges and in resolving the issues of omission and misdetection that affect the accuracy of position information. Simultaneously, the algorithm must possess exceptional real-time performance in order to satisfy the UAV’s real-time inspection requirements. Therefore, while ensuring real-time performance, we optimized the model primarily to improve segmentation accuracy, reduce fluctuations in positional information caused by missed detections, and enhance inspection stability.

Consequently, we suggest the WaterSegLite (Water Segmentation Lightweight algorithm), a water segmentation model that is both efficient and lightweight and is improved by LiteSeg (a novel lightweight convnet for semantic segmentation). This algorithm can rapidly segment water bodies to produce mask images, thereby establishing a foundation for the subsequent extraction of location information. MobileNetV2-S (MobileNetV2-Small), a compact version of the MobileNetV2 neural network, is the initial step in this algorithm. Designed for mobile and embedded devices, MobileNetV2 is a lightweight convolutional neural network that is extensively employed. In our method, the network’s real-time performance is significantly enhanced, and computational costs are significantly reduced by applying early-stage downsampling to only the first eight layers of MobileNetV2. The DP-ASPP module (Dense Parallel Atrous Spatial Pyramid Pooling) is subsequently introduced to further improve the network’s receptive field. The DP-ASPP network captures richer contextual information from aerial images and ensures the effective extraction of water body boundaries by utilizing dense connectivity within a multi-scale pyramid structure. This design achieves a balance between computational efficiency and the expansion of the receptive field by maintaining a consistent dilation rate. Finally, a lightweight attention mechanism is implemented to improve the network’s capacity to extract fine-grained edge features, such as water boundaries. The CPG (Carrot in Probabilistic Grid) algorithm is employed in the visual navigation component to generate forward navigation directions for unmanned aerial vehicles (UAVs) over waterbodies that resemble rivers. A tracking algorithm, CPG, is particularly well-suited for path planning in linear water bodies, such as rivers, as it determines the tracking direction using a probabilistic grid. GPS (Global Positioning System) information is utilized to enable comprehensive UAV inspections of lake-like water bodies.

This investigation suggests a real-time water region segmentation algorithm that is both lightweight and efficient, with an emphasis on the utilization of small unmanned aerial vehicles (UAVs) and edge computing devices for water surface inspection. The unique contributions of this study are as follows: (1) the development of a segmentation model that is both highly accurate and real-time, optimized for small edge devices, in contrast to the conventional solution that depends on large UAVs and high-performance computing devices; (2) the proposed inspection method for rivers and lakes, which improves the algorithm’s adaptability and flexibility; (3) the practical deployment of this method on a small UAV platform, which significantly reduces the hardware cost and offers a more practical solution for the widespread application of UAV inspection in ecological environment management.

Our work is presented as follows: Section 2 describes the methodology used as well as the experimental conditions; Section 3 presents the experimental results; and Section 4 discusses the conclusion.

## 2. Materials and Methods

This chapter describes the water region segmentation method, the visual navigation of UAVs through water region segmentation, and the experimental conditions and evaluation metrics of the experiment.

### 2.1. Drone Segmentation of Riverbank Line Methods

Taking WaterSegLite as the foundational framework and integrating MobileNetV2-S, DP-ASPP, and ECA (Efficient Channel Attention), we propose a new fast semantic segmentation network to address the challenge of balancing real-time processing and segmentation accuracy in unmanned aerial vehicle (UAV) water inspection algorithms. The lightweight computer vision architecture MobileNetV2-S is used as the backbone network, which greatly reduces the computational overhead of the model while extracting features efficiently. In order to address the problem of boundary leakage and misdetection in water segmentation, the DP-ASP is used to expand the receptive field to reduce the misdetection of the water boundary by combining the high-resolution characteristics of the UAV aerial viewpoint. In addition, the feature representation is further optimized by using the ECA attention mechanism, which can capture the subtle differences of the water boundary region more accurately and enhance the segmentation ability of the model for the water body. The designed novel network can effectively extract multi-scale features from aerial images, capture the details of the water boundary more accurately, and significantly improve the segmentation performance. A balance between lightweight design and high accuracy is achieved, providing an innovative solution for resource-constrained edge devices to meet the needs of UAV watershed inspection.

**Figure 1:**
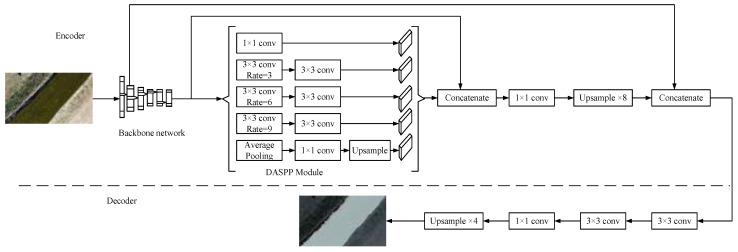
LiteSeg network architecture.

Figure 1 illustrates the LiteSeg network structure, which is divided into two components: the encoder structure and the decoder structure. The backbone network is employed in the encoder structure to extract image features. The DASPP (Dual Atrous Spatial Pyramid Pooling) module is fed the high-level features for multi-scale feature extraction. The short-range skip connections connect the high-level features with the DASPP output, while the long-range skip connections connect the end of the encoder structure with the shallow features. The first layer in DASPP employs parallel dilated convolution with dilation rates of 1, 3, 6, and 9, while the second layer employs standard 3 × 3 convolution. The decoder structure employs two 3 × 3 convolution layers to process the features and a 1 × 1 convolution to reduce the number of low-level feature channels. A 4× upsampling operation is used to restore the output features to the original image size.

#### 2.1.1. MobileNetV2-S

The MobileNetV2 backbone network is optimized in this paper to enhance the algorithm’s real-time detection of the rapidly changing aquatic environment during UAV inspections. The initial layers of the MobileNetV2 network structure are primarily concerned with the extraction of spatial and textural features, which are particularly crucial for the precise delineation of boundaries in water body segmentation tasks. The flight altitude and river width will fluctuate during the inspection task; however, the textural characteristics of the water regions will be markedly distinct from those of the background. Therefore, in order to maintain the recognition capability for water body boundaries to the greatest extent possible, the texture feature extraction capability of the first eight layers is needed. The final three layers, which are designed for tasks such as object detection and classification, are redundant for the water region segmentation task due to their inability to meet the needs of spatial feature preservation and pixel-by-pixel prediction. Simultaneously, the computational volume is substantially increased by the substantial increase in channel count to 1280, which occurs from the ninth layer. Consequently, we maintain the initial eight layers of the network to enhance the model’s running efficiency and guarantee the feature extraction capability. The step configuration of the network structure is optimized by setting the step of the convolution unit in the sixth layer to 2 and adjusting it to 1 in the seventh layer. This process advances the downsampling operation of the bottleneck module in the seventh layer and reduces the size of the feature map earlier. This reduces the computational overhead of the subsequent layers while maintaining the feature representation capability. 

Table 1 shows the MobileNetV2 network architecture. Table 2 illustrates the layers of the MobileNetV2-S network. The number of output channels is denoted by “c”, the layer number is denoted by “layer”, the number of “bottleneck” repetitions is denoted by “*n*”, the expansion factor is denoted by “t”, and the step size is identified by “s”, “–” means no such parameter for this layer.

#### 2.1.2. DP-ASPP

Larger receptive fields facilitate the model’s ability to capture the global morphology of the target river in high-resolution images from the UAV viewpoint. Additionally, the model’s capacity to identify features at the river boundary is enhanced by the integration of a broader range of contextual information, which mitigates the issue of mis-segmentation during the detection process. ASPP employs parallel dilated convolutions with varying dilation rates to capture multi-scale contextual information. However, as the dilation rate is increased, the receptive field may become enlarged, resulting in sparse sampling by the convolutional kernel and the loss of critical local features. We suggest DP-ASPP, which is inspired by DenseASPP (Dense Atrous Spatial Pyramid Pooling) [22]. This approach samples the outputs of dilated convolutions in a densely connected manner to fuse the outputs of the dilated convolutions. This method allows the model to achieve larger receptive fields without necessitating high dilation rates while simultaneously addressing the feature degradation issue that is caused by dilated convolutions at high dilation rates. In a manner similar to Res2Net-Seg [23], we integrate the pooling features into the multi-scale fusion process. Nevertheless, we integrate feature maps through concatenation, as opposed to convolution operations, in contrast to Res2Net-Seg. This design decision aids in the preservation of river boundary information and minimizes the loss of fine details.

The receptive field of the network is expanded by dilated convolution, which permits the extraction of larger-scale features without incurring additional computational costs, assuming the original image resolution. In the ASPP, the maximum receptive field for dilated convolution is 19, depending on the aggregation of multi-scale contextual information.

(1) $$R_{n}=dK_{n}-d+1=19$$

where “d” is the dilation rate, “$K_{n}$” is the size of the convolution kernel, and “$R_{n}$” is the size of the receptive field. Parallel dilated convolution is implemented with dilation rates of 1, 3, 6, and 9. The maximum receptive field is the one that corresponds to a dilation rate of 9.

In the DP-ASPP network, each layer of dilated convolution is preceded by a mechanism where each layer receives feature maps from all preceding layers as additional inputs. At the same effective dilation rates as ASPP, the maximum receptive field is 37.

(2) $$R_{max}=k_{3d=3}+k_{3d=6}+k_{3d=9}-2=37$$

**Figure 2:**
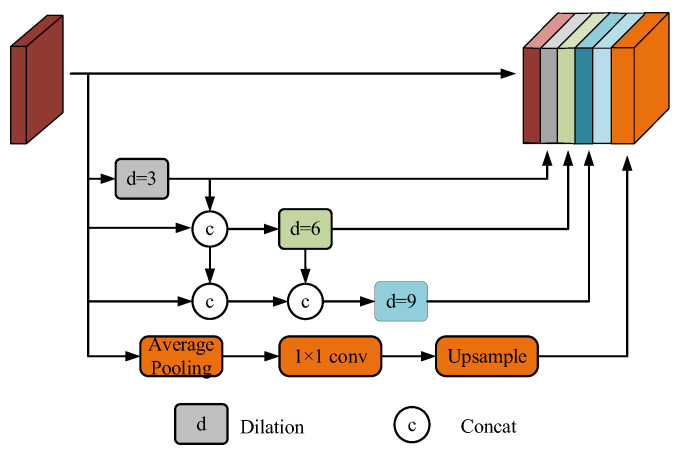
DP-ASPP network structure.

Compared to dilation convolution for multi-scale contextual information aggregation, the DP-ASPP receptive field size has increased by 18. DP-ASPP retains the original global average pooling structure while using dense connections, which can better capture global information when dealing with water body images from an aerial viewpoint, reduce the loss of detailed features during information transfer, and at the same time promote the flow of information between different layers, which improves the model’s expression ability. The DP-ASPP network structure is shown in Figure 2.

#### 2.1.3. Introduction of the ECA Attention Mechanism

In the process of UAV inspection, the accurate identification of the river boundary is crucial and affects whether the UAV can accurately track the target area, so we introduce an attention mechanism into the algorithm to better identify the edge features. Although the traditional attention mechanism can effectively enhance the model’s attention to important features, it is often accompanied by a significant increase in the number of parameters, which affects the model’s ability to detect the waterways in real time.

**Figure 3:**
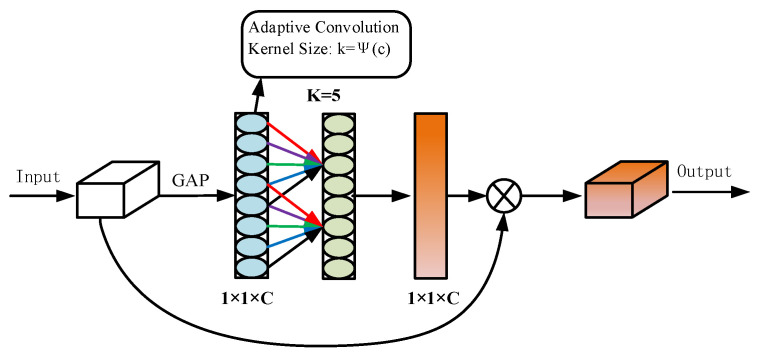
ECA attention mechanism network structure diagram.

**Figure 4:**
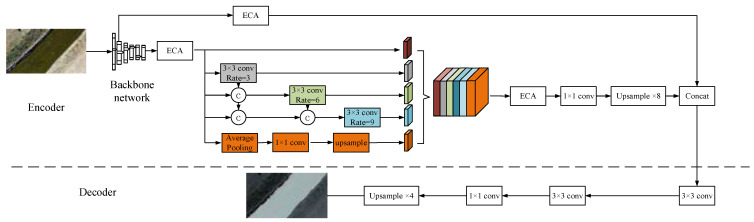
The WaterSegLite network.

In order to resolve this contradiction, the ECA [24] attention mechanism is implemented. Figure 3 illustrates this. ECA is capable of determining the channel importance of the feature map in a straightforward and efficient manner by employing a global average pooling operation. Consequently, one-dimensional convolution is employed to simulate the relationship between the channels without significantly increasing the computational effort, thereby adjusting the weights of each channel. The model differentially handles different feature channels and prevents the loss of important information during feature dimensionality reduction by employing the adaptive adjustment strategy of the convolution kernel size *k* in Equations (3) and (4). In this manner, the ECA attention mechanism more effectively captures the cross-channel dependencies, improves the response to boundary features, and decreases the incidence of missegmentation and missed segmentation. The WaterSegLite network structure presented in Figure 4.

(3) $$C=2^{\left(\gamma *k-\beta \right)}$$

(4) $$k=\psi \left(C\right)={\left|\frac{\mathrm{lo}g_{2}\left(C\right)}{\gamma }+\frac{b}{\gamma }\right|}_{\mathrm{odd}}$$

where “*k*” denotes the one-dimensional convolutional kernel size, “*C*” denotes the input feature channel dimension, “*γ*” denotes the exponential function exponential linear mapping coefficients, “*β*” denotes the exponential function exponential linear mapping constant, and “*odd*” denotes the closest odd number.

### 2.2. UAV Water Surface Tracking Methods

**Figure 5:**
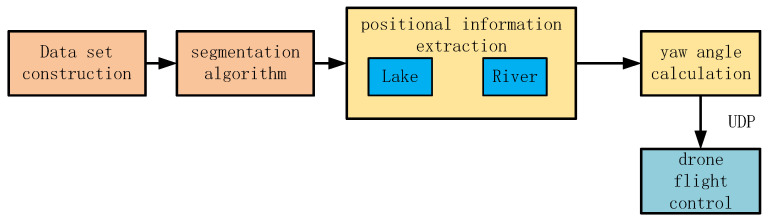
Visual navigation scheme.

We deployed the trained WaterSegLite on edge devices. After the algorithm segments the water area mask, we formulate corresponding navigation plans based on different types of water bodies, such as rivers and lakes, and extract navigation positional information. Subsequently, the positional information is used to calculate the yaw angle of the drone. The yaw angle information is sent from the algorithm end to the flight control end via the UDP protocol to control the flight attitude of the drone. Figure 5 illustrates the visual navigation scheme.

#### 2.2.1. Drone River Tracking

In order to provide the UAV with a direction to fly, the river must be tracked based on the river mask obtained through semantic segmentation between consecutive frames. The Carrot in Probabilistic Grid (CPG) algorithm [25] is employed to direct the UAVs to follow line segments in a specific direction. This process creates a grid-segmented image in the UAV’s aerial view and establishes a target location for chasing. We optimize the CPG algorithm using the area characteristics of rivers, which are distinguished from line segments by their complex shapes and large widths.

The specific tracking process for river patrol is as follows:

1. Calculate the grid probability as shown in Equations (5) and (6).
  (5) $$A=\Sigma_{i=1}^{5}a\left(G_{i}\right)$$
  (6) $$p\left(G_{i}\right)=a\left(G_{i}\right)/A$$
  where “$a\left(G_{i}\right)$” denotes the area of the river mask in the $G_{i}$ grid, “A” denotes the sum of the areas of all grids except $G_{0}$, and “p($G_{i}$)” denotes the probability of occurrence of the $G_{i}$ grid. Here, only the first six grids are taken for determining the UAV’s forward direction.
2. Extract the maximal mesh Gr and calculate the maximum mesh center of mass.
  (7) $$M_{00}=\sum_{i}\;\sum_{j}\;V\left(i,j\right)$$
  (8) $$M_{10}=\sum_{i}\;\sum_{j}\;i\cdot V\left(i,j\right)$$
  (9) $$M_{01}=\sum_{i}\;\sum_{j}\;j\cdot V\left(i,j\right)$$
  (10) $$x_{c}=\frac{M_{10}}{M_{00}}+w\;\;y_{c}=\frac{M_{01}}{M_{00}}+h$$
  where “$V\left(i,j\right)$” is the pixel value of the binary image at “$\left(i,j\right)$”. “$M_{00}$” is the zero-order moment of the image, and “$M_{10}$” and “$M_{01}$” are the first-order moments of the image. “$x_{c}$”, “$y_{c}$” are the coordinates of the center of mass. “w”, “h” are determined by the maximal grid, “$G_{r}$”. For example, if the maximal grid is “$G_{4}$” under the size of the 640 × 360 image, “w”, “h” are both 0. If the maximal grid is “$G_{1}$”, then “w” is 640 × 2/3 and “h” is 120.
3. Determine the motion command.
  (11) $$\mathrm{angle}=arcta\;n\frac{320-x_{c}}{160-y_{c}+1\times 10^{-10}}$$
  (12) angle=angle×180/π
  (13) $$yaw=\left\{\begin{array}{c} 90^{\circ }+angle,\;angle<0\\ angle-90^{\circ },\;angle>0 \end{array}\right.$$

The radian-to-degree formula is represented by Equation (12), while Equation (13) calculates the yaw angle of the UAV. The yaw angle is negative when the center of mass coordinate is in the first and fourth quadrants and positive when it is in the second and third quadrants. The yaw angle is used to correct the UAV’s heading and determine its direction of advancement. The center point of the image pixel is at (160, 320), which is the same as the center of mass of the UAV aerial camera when the coordinate system is converted with the UAV.

**Figure 6:**
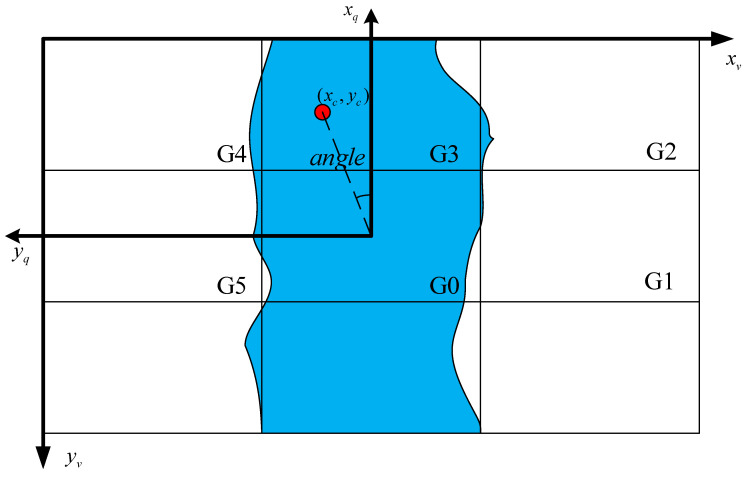
UAV river visual navigation diagram.

The UAV River vision navigation schematic is shown in Figure 6, where “$x_{v}$” and “$y_{c}$” are in the 2D image coordinate system, “$x_{q}$” and “$y_{q}$” represent the 2D coordinate system of the UAV relative to the image, and “$x_{q}$” corresponds to the positive direction of the UAV nose.

**Figure 7:**
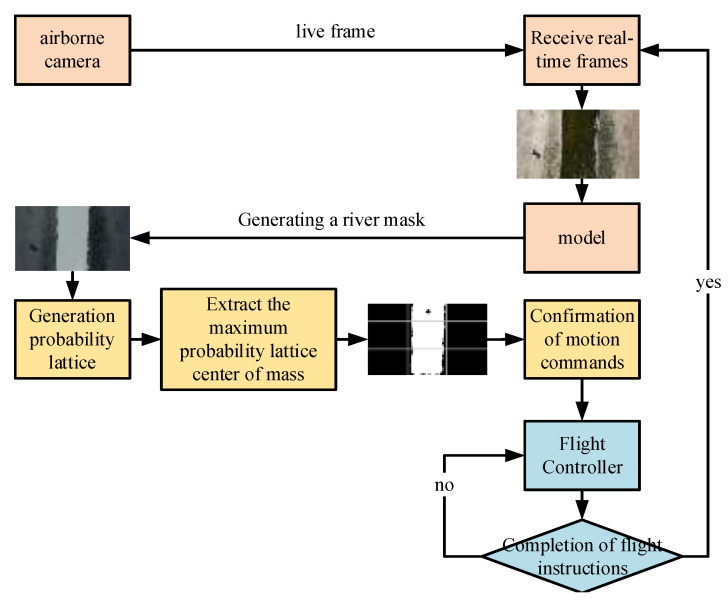
Flow chart of UAV river inspection.

The UAV river inspection flowchart is shown in Figure 7. The onboard camera captures aerial images of the river, which are processed by the WaterSegLite algorithm to generate the river mask. The CPG algorithm then computes the UAV’s forward movement direction based on the river mask. Finally, the flight controller executes motion commands to guide the UAV in completing the river inspection.

#### 2.2.2. Drone Lake Inspection

**Figure 8:**
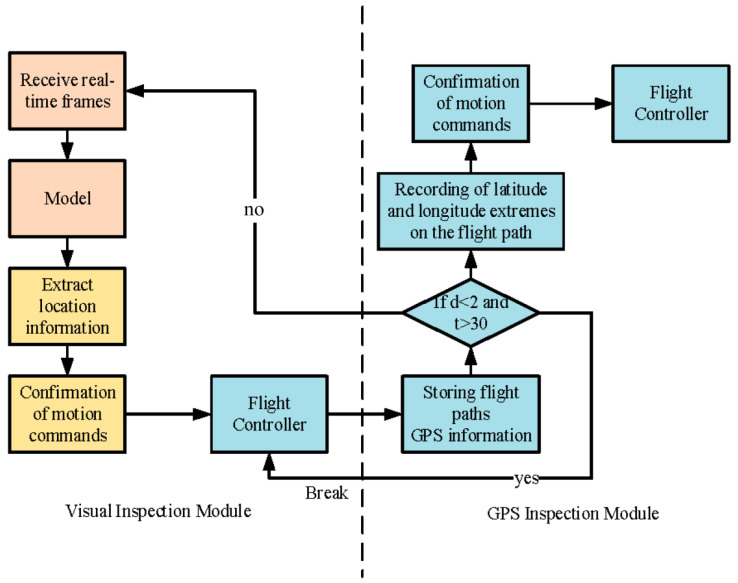
Flow chart of UAV lake inspection.

The inspection range of the CPG algorithm is restricted to rivers within the image range. When confronted with lakes with a larger range, our inspection strategy is to first inspect the edges of the lake and subsequently complete the inspection of the entire lake using the latitude and longitude information on the UAV’s flight path. Figure 8 illustrates the flowchart for the UAV lake inspection. Initially, the semantic segmentation algorithm is employed to inspect the lake’s edge. Subsequently, the flight controller is employed to record the latitude and longitude extremes on the flight trajectory. Finally, serpentine inspection is conducted within the range of latitude and longitude extremes to guarantee that the entire lake is inspected. When the takeoff time is more than 30 s later than the current position of the UAV, the distance between the two is less than 2 m. This is considered the end of the UAV lake edge inspection, the end of the visual navigation inspection, and the beginning of the GPS inspection. If the distance is greater than 2 m, the flight trajectory information is always recorded.

**Figure 9:**
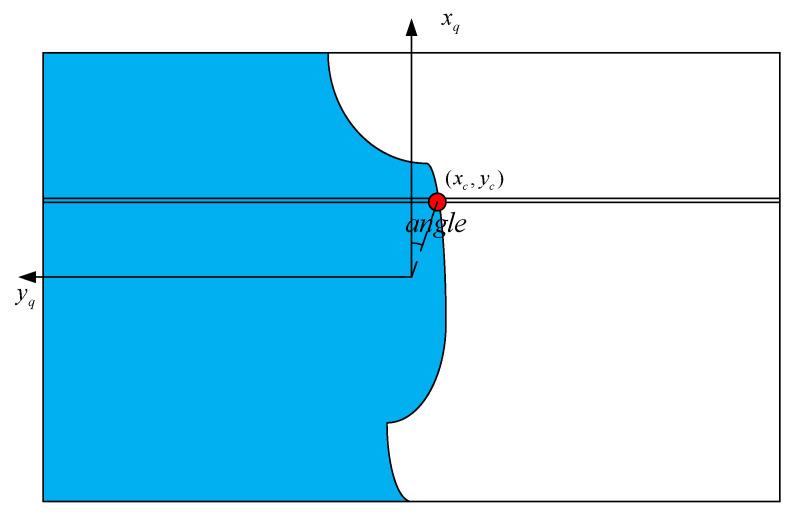
Visual navigation diagram of lake for UAV.

Lake edge inspection requires extracting information about the location of lake boundary points by shifting one pixel point up and one pixel point down along the vertical direction in one-third of the image to define a local region of interest. Within this local region, the contour of the image is extracted using the contour detection algorithms. After extracting the contour, the rightmost contour point is determined by traversing all contour points in the region, which is the boundary point of the lake “*x_c_*” and “*y_c_*”. The corresponding yaw angle is computed as shown in Equation (13). Figure 9 shows the Visual navigation diagram of lake for UAV.

### 2.3. Materials

#### 2.3.1. Dataset Production

**Figure 10:**
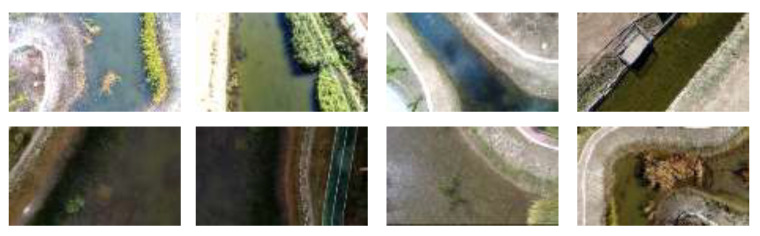
Images of different types of watershed datasets.

The dataset was primarily collected from rivers and lakes at different altitudes, lighting conditions, and weather using UAV aerial photography. The camera is positioned vertically downward, and the captured video is saved at a resolution of 1980 × 1020, with one image per frame. The UAVs fly at 10 m, 15 m, and 20 m. The dataset is expanded by employing six data enhancement methods, including luminance enhancement, contrast enhancement, image flipping, image rotating, HSV enhancement, and affine transformation, to guarantee the generalizability of the model. The final total is 1500 images. Fine labeling is accomplished through the utilization of the open-source annotation software LabelMe, with the training and validation sets separated by a 9:1 ratio. Figure 10 shows the type of dataset.

#### 2.3.2. Experimental Environment and Evaluation Indicators

This experiment was conducted on a workstation that was equipped with the Ubuntu 18.04 operating system, PyCharm (2023.3) development software, an NVIDIA GeForce RTX 3090 Ti (Intel, Santa Clara, CA, USA) graphics card model, and an Intel Core i7-12700K processor (Intel, Santa Clara, CA, USA). After completing the model training, we deployed WaterSegLite on the edge device NVIDIA Jetson Xavier NX (Intel, Santa Clara, CA, USA) using the PyTorch (1.9.1) framework. The environment required for WaterSegLite was set up on the edge device, and the model weights trained on the workstation were transferred to the edge device.

In this paper, the training image size is 512 × 512 pixels, the batch size is set to 16, and 150 iterations are performed to ensure that the model fully converges. The initial learning rate is initialized to 0.001, and the learning rate decreases by 0.0005 every five rounds, using the SGD optimizer with momentum set to 0.9.

Evaluation metrics are used to assess the segmentation effect of the model; commonly used are pixel accuracy (PA), mean intersection over union (mIoU), recall (Recall), and mean pixel accuracy (MPA). In this paper, we use the mIoU and F1 value as the metrics for assessing the segmentation effect of the modeled water shoreline. mIoU is used to measure the similarity of the segmentation result to the real annotation, and it is expressed by the formula as in Equation (14).

(14) $$mIOU=\frac{1}{k+1}\sum_{i=0}^{k}\;\frac{TP}{FN+FP+TP}$$

where “k + 1” represents the number of categories (in this paper, the number of categories is 2 for water surface and backgrounds; thus, “k + 1” is set to 2). The number of pixels that were correctly classified as water surface is denoted by “TP”, while the number of pixels that were predicted as background is denoted by “FN”. Additionally, the number of pixels that predicted backgrounds as water surface is marked by “FP”.

The formulas for accuracy and recall are expressed as Equations (15) and (16).

(15) $$Precision=\frac{TP}{TP+FP}$$

(16) $$Recall=\frac{TP}{TP+FN}$$

There is often a trade-off between precision and recall; when recall is high, precision tends to be low, and vice versa. In order to comprehensively evaluate these two important metrics, the F1 score is introduced, which is the harmonic mean of precision and recall, and the larger the F1 value, the better the comprehensive performance of the model in the classification task and the better the segmentation effect. The formula is given in Equation (17).

(17) $$F1=\frac{2\times P\times R}{P+R}$$

The proposed WaterSegLite adopts a MobileNetV2-S backbone with a tailored DP-ASPP module, optimized for UAV navigation in water regions. This architecture effectively balances computational efficiency and segmentation accuracy while maintaining robustness in high-resolution UAV imagery. Unlike conventional semantic segmentation models, WaterSegLite achieves superior performance in real-time applications, particularly in terms of segmentation speed, accuracy, and computational resource efficiency, rendering it an ideal solution for UAV systems. The following sections assess the performance and effectiveness of the proposed method through comparative experiments and diverse task-specific evaluation metrics.

## 3. Results

In this chapter, the backbone network comparisons, ablation studies, and error analyses for the water region segmentation algorithm are conducted. Comparative experiments with other models and experiments with public datasets are also conducted.

### 3.1. Backbone Network Comparison Experiment

In order to verify the effectiveness of the backbone network improvement, we conduct backbone network comparison experiments using DarkNet, MobileNetV2, ShuffNet, and MobileNetV2-S to compare the differences in the mean Intersection over Union (mIoU) ratio, as well as the number of parameters and the frame rate of different backbone networks.

Table 3 illustrates that the DarkNet network is incapable of meeting the demands of real-time inspection as a result of the excessive number of parameters, despite its exceptional segmentation capabilities. In contrast, MobileNetV2-S maintains a sufficient segmentation capability while achieving the highest real-time frame rate of 312 and a mIoU ratio of 90.80. This illustrates the potential of the MobileNetV2-S for the real-time segmentation of water bodies for UAVs.

### 3.2. River Dataset Ablation Experiments

In order to verify the effects of MobileNetV2-S, DP-ASPP, and the addition of the ECA attention mechanism on the model segmentation effectiveness, WaterSegLite sets up different comparison experiments. The effects of the different improvements on the mIoU ratio, F1 value, number of parameters, number of FLOPs, and running speed of the model are compared and validated on the dataset in the same deep learning environment, and the results are shown in Table 4, “√”indicates the use this module.

Table 4 illustrates that the mIoU and F1 values of the Group 2 MobileNetV2-S backbone network are reduced by 0.31% and 0.01%, respectively, and the floating-point operations (FLOPs) and parameter counts are reduced by 1.02 and 2.27, respectively. The frame rate per second is increased by 19, which significantly improves the model’s processing speed, despite a slight decrease in the average intersection ratio. In comparison to the model with only MobileNetV2-S, the addition of DP-ASPP improves the mIoU by 0.96%, the F1 value decreases by 0.01%, the number of FLOPs and parameters decreases by 0.02 and 0.03, and the frame rate per second decreases by 0.99. Comparing Groups 3 and 4, the addition of the ECA attention mechanism improves the model processing speed by 0.99 compared to the model with only MobileNetV2-S and using the DP-ASPP module, the model mIoU, F1 is improved by 2.05%, 2.09% the number of floating points and the number of parameters remain unchanged, and the frame rate per second decreases by 17, which shows that the ECA attention mechanism does not increase the computational amount of the model, and the effect of the enhancement of the model mIoU is better based on it; comparing Scenario 1 and Group 4, compared to the original LiteSeg semantic segmentation network model, the WaterSegLite mIoU is improved by 2.7%, the F1 value is improved by 2.23%, the number of FLOPs and the number of parameters are decreased by 1.04 and 2.3, respectively, and the frame rate of the model per second is improved by 1.36 frames per second, and the WaterSegLite significantly enhances the segmentation accuracy of the model without affecting the real-time speed, and it can reach 28.27 frames per second on the airborne equipment which meets the demand of real-time inspection.

**Figure 11:**
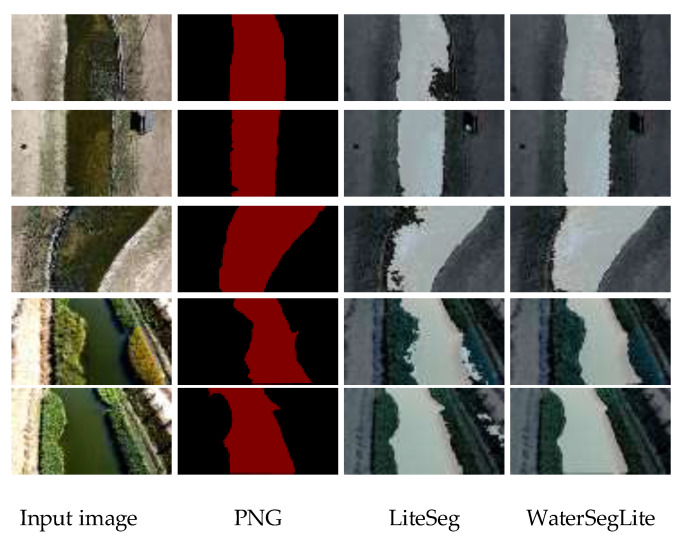
Comparison of model segmentation results.

The segmentation results of the WaterSegLite are more accurate than those of the initial LiteSeg algorithm, as evidenced by the comparison of the improved and initial models in Figure 11. This improvement in the ability to extract the feature information of the water shoreline is a result of the reduced leakage and incorrect segmentation phenomena. The WaterSegLite’s segmentation mask is more closely aligned with the actual water shoreline during the flight experiment. Consequently, the extracted position information will be more accurate and stable, and the number of incorrect position information due to missed segmentation and incorrect segmentation will be reduced. This will result in a more stable water surface inspection for the UAV.

### 3.3. Error Analysis

**Figure 12:**
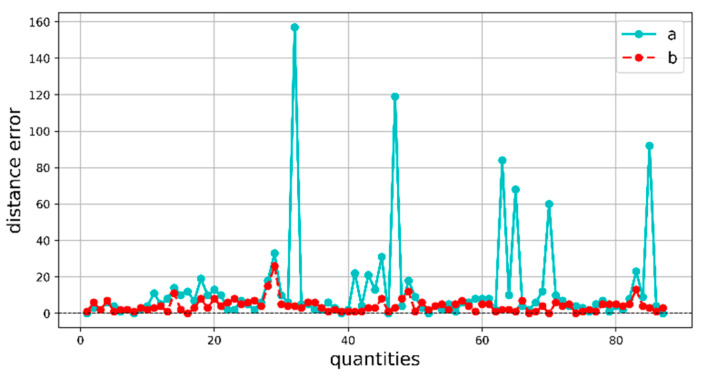
Comparison plot of model position information error.

To verify the accuracy of location information extracted by WaterSegLite for river tracking, we employ the boundary pixel coordinates extracted from the manually labeled river data mask images as the ground truth. The original algorithm and the improved algorithm are subsequently subjected to the same location information extraction operation, resulting in the recording of the original and improved values, respectively. We also extract location information for lakes and compare the corresponding visualizations. Figure 12 shows the Comparison plot of model position information error.

The difference between the original value and the true value is represented by curve “a” in the figure, while the difference between the improved value and the true value is represented by curve “b”. The error between the manually labeled real water shoreline location information and the position information of the water shoreline extracted by the original and improved models is represented by the two curves. The location information extracted by the WaterSegLite is more accurate, as evidenced by the figure. We conducted an analysis of several points where the LiteSeg error fluctuates more and discovered that the model misdetected the grass outside the boundary, whereas the WaterSegLite effectively mitigates this misdetection.

### 3.4. Comparative Experiments with Different Models

In order to verify the performance of the improved backbone network, Bisenet [26], DDRNet-23 [27], SwiftNet [28], PIDNet [29], Enet [30], and Espnet [31] are selected. Bisenet effectively reduces the computational cost and improves the real-time performance through its unique two-branch structure, where one branch is responsible for capturing high-resolution spatial features and the other branch captures contextual information and synthesizes the advantages of the two through the feature fusion module, thus realizing efficient and high-precision segmentation algorithms suitable for fast-changing water scenarios. ENet gradually reduces the resolution of the aerial images through multiple downsampling and adopts the factorization convolution method in the convolutional layer, which significantly reduces the number of model parameters and computational requirements, making it suitable for application in the water body segmentation task with high real-time requirements. Espnet effectively reduces the number of channels by point-by-point convolution and at the same time, larger receptive fields by introducing the dilated convolution so as to enable efficient operation of Espnet in the onboard equipment with limited resources. DDRNet adopts a dual-resolution network design, which concurrently extracts high-resolution, detailed spatial features and low-resolution, global contextual information. This architecture facilitates computationally efficient semantic segmentation while achieving precise boundary localization. SwiftNet employs a lightweight ResNet-based encoder combined with an optimized decoder module, enabling real-time semantic segmentation with high accuracy and computational efficiency. PIDNet is specifically designed for efficient real-time semantic segmentation, incorporating a multi-branch network architecture to achieve accurate segmentation of primary objects and detailed boundary refinement while maintaining a favorable trade-off between inference speed and accuracy. The experimental results are presented in Table 5.

From Table 5, the experimental results highlight the strengths of various models in terms of mIoU, parameter size, and FPS, showcasing their suitability for different applications. Among them, WaterSegLite achieves the highest mIoU of 93.81% with only 2.08 M parameters and an FPS of 294, making it the most suitable for real-time water inspection tasks where both accuracy and efficiency are critical. Compared to other models, WaterSegLite provides an optimal balance between lightweight design and segmentation precision, outperforming both ENet and EspNet in accuracy while maintaining a comparable parameter size and offering higher efficiency than models like DDRNet. This makes WaterSegLite the ideal choice for resource-constrained scenarios in dynamic and complex water environments.

### 3.5. Dataset Generalization Experiment

**Figure 13:**
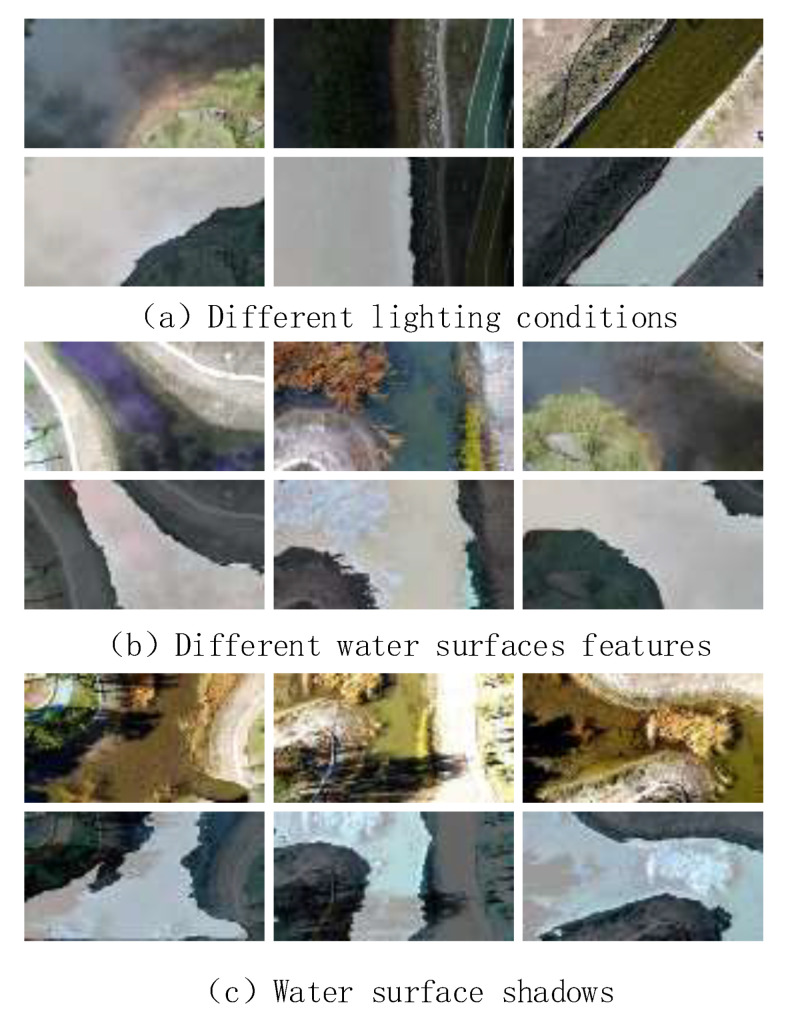
Model segmentation effect under different conditions.

To discuss the impact of challenges such as lighting variations, different water surface features, and water surface shadows on segmentation results, we have incorporated a generalization experiment to evaluate the robustness and adaptability of the model under these varying conditions. Figure 13 illustrates the segmentation results of the model under different conditions, clearly demonstrating the model’s performance across these scenarios. By comparing these results, we can observe the accuracy of the model’s segmentation, providing a broader range of environmental conditions for practical drone inspections.

### 3.6. Comparison of AFID Dataset Model Improvements

The original LiteSeg algorithm achieves good results on the Cityscapes dataset, but the rivers that are the subject of this paper are not adequately represented in this dataset. In order to verify the validity of the model, this paper performs a comparison on the AFID [32] dataset. We use the top-down view from UAV imagery of “All” in the AFID dataset to simulate the detection of rivers by the model during drone inspections. Comparative experiments are conducted with models DDRNet, PIDNet, and SANet [33]; the experimental results are shown in Table 6.

In the results, we comprehensively verify the performance advantages and practicality of the proposed algorithm through a series of experiments. The backbone network comparisons and ablation studies show that the designed WaterSegLite model achieves a good balance between accuracy and efficiency, while the optimized model significantly improves the water region segmentation accuracy. Comparison experiments with other models further demonstrate the advantages of the method in terms of lightweight and real-time performance, and experiments on error analysis of UAV position information show that the segmentation results can effectively support UAV navigation tasks. In addition, experiments on the AFID dataset verify the reliability of the algorithm. Compared with PIDNet, the parameter count and floating-point computation are significantly reduced, the frame rate is notably improved, and resource consumption is minimized. Taken together, WaterSegLite has efficient and accurate segmentation capabilities and provides a robust and practical solution for UAV navigation in water scenarios.

## 4. Conclusions

Traditional manual inspection methods are costly and inefficient. The advantages of UAVs, such as high mobility and wide coverage, make them suitable for aerial photography inspection of waters. Therefore, based on a small UAV, we designed the idea of inspecting two types of waters, lakes and rivers, and proposed a water segmentation algorithm—WaterSegLite, which extracts the corresponding position information through the characteristics of the segmentation mask and adjusts the UAV flight attitude to complete the visual navigation. In the research of the water segmentation algorithm, we constructed the UAV aerial water data set of rivers and lakes and other water types and carried out an in-depth study on the position information jumping problem caused by the wrong segmentation and omission of segmentation of water edges by the baseline model, as well as the balance between segmentation effect and real-time performance, and optimized the network structure of the baseline model so as to make the model able to better segment the boundary of the water and to meet the high processing speed of UAV in real-time flight. By optimizing the network structure of the baseline model, the WaterSegLite can better segment the water boundary and meet the high processing speed of the UAV in real time. Finally, the segmentation accuracy (mIoU) of WaterSegLite reaches 93.81%, and the F1 value reaches 95.44%, which are 2.7% and 2.23% higher than that of the baseline model, respectively. Meanwhile, the processing frame rate of the algorithm on the airborne device reaches 28.27 frames/s. In real-time inspection, the algorithm can quickly and accurately segment the water boundary and extract the position information to adjust the UAV’s flight attitude in time. Our method provides technical support for UAV inspection in the water environment, but the strong reflection of the water surface and the complex water environment still need to be dealt with, and future research will continue to optimize the dataset to improve the processing ability of the model in the complex water environment and the stability of the inspection. Meanwhile, consider adding additional sensors (e.g., LiDAR, radar) to complement visual navigation, attempts will also be made to establish the connection between water flow and UAV attitude [34], as well as to capture the dynamics of floating objects in water [35].

## Figures and Tables

**Table 1:** MobileNetV2 network architecture.

Layer	Operator	c	*n*	t	s
1	conv2d	32	1	–	2
2	bottleneck	16	1	1	1
3	bottleneck	24	2	6	2
4	bottleneck	32	3	6	2
5	bottleneck	64	4	6	2
6	bottleneck	96	3	6	2
7	bottleneck	160	3	6	1
8	bottleneck	320	1	6	1
9	conv2d 1 × 1	1280	1	–	1
10	avgpool 7 × 7	–	1	–	–
11	conv2d 1 × 1	k	–	–	–

**Table 2:** MobileNetV2-S network architecture.

Layer	Operator	c	*n*	t	s
1	conv2d	32	1	–	2
2	bottleneck	16	1	1	1
3	bottleneck	24	2	6	2
4	bottleneck	32	3	6	2
5	bottleneck	64	4	6	2
6	bottleneck	96	3	6	2
7	bottleneck	160	3	6	1
8	bottleneck	320	1	6	1

**Table 3:** Backbone network comparison experiment.

Network	Params	FPS (360×640)	mIoU%
DarkNet	20.55	182	91.89
ShuffNet	3.51	246	90.06
MobileNetV2	4.38	293	91.11
MobileNetV2-S	2.11	312	90.80

**Table 4:** The results of the ablation experiment were compared.

Group	MobileNetV2-S	DP-ASPP	ECA	mIoU%	F1%	GFLOPS	Params	FPS
1				91.11	93.21	4.9	4.38	293
2	√			90.80	93.20	3.88	2.11	312
3	√	√		91.76	93.35	3.86	2.08	311
4	√	√	√	93.81	95.44	3.86	2.08	294

**Table 5:** Comparative experiments of different lightweight semantic segmentation models.

Model	Network	mIoU%	Params	FPS
Bisenet	Resnet18	91.41	49.0	220
ENet	None	88.92	0.37	306
Espnet	None	89.33	0.36	277
LiteSeg	MobileNetV2	91.11	4.38	293
PIDNet	PID	93.65	22.28	265
SwiftNet	Resnet18	92.5	11.8	261
DDRNet	Dual Resolution	93.34	30.86	245
WaterSegLite	MobileNetV2-S	93.81	2.08	294

**Table 6:** Comparative experiments on public datasets.

Model	mIoU%	F1%	Params	GFLOPs	FPS
LiteSeg	88.48	90.85	4.38	4.96	293
DDRNet	89.57	91.26	30.86	35.26	245
PIDNet	90.21	91.9	27.21	22.28	265
SANet	88.95	91.08	7.88	8.77	253
WaterSegLite	90.00	91.56	2.08	4.08	294

## Data Availability

All data used in this paper can be obtained by contacting the authors of this study.
